# Brain Tumor Segmentation and Classification from Sensor-Based Portable Microwave Brain Imaging System Using Lightweight Deep Learning Models

**DOI:** 10.3390/bios13030302

**Published:** 2023-02-21

**Authors:** Amran Hossain, Mohammad Tariqul Islam, Tawsifur Rahman, Muhammad E. H. Chowdhury, Anas Tahir, Serkan Kiranyaz, Kamarulzaman Mat, Gan Kok Beng, Mohamed S. Soliman

**Affiliations:** 1 Centre for Advanced Electronic and Communication Engineering, Department of Electrical, Electronic and Systems Engineering, Faculty of Engineering and Built Environment, Universiti Kebangsaan Malaysia, Bangi 43600, Malaysia; 2 Department of Computer Science and Engineering, Dhaka University of Engineering and Technology, Gazipur, Gazipur 1707, Bangladesh; 3 Department of Electrical Engineering, Qatar University, Doha 2713, Qatar; 4 Department of Electrical, Electronic and Systems Engineering, Faculty of Engineering and Built Environment, Universiti Kebangsaan Malaysia, Bangi 43600, Malaysia; 5 Department of Electrical Engineering, College of Engineering, Taif University, P.O. Box 11099, Taif 21944, Saudi Arabia; 6 Department of Electrical Engineering, Faculty of Energy Engineering, Aswan University, Aswan 81528, Egypt

**Keywords:** brain tumor segmentation, classification, antenna sensor, deep learning, Self-ONN, sensor-based microwave brain imaging system

## Abstract

Automated brain tumor segmentation from reconstructed microwave (RMW) brain images and image classification is essential for the investigation and monitoring of the progression of brain disease. The manual detection, classification, and segmentation of tumors are extremely time-consuming but crucial tasks due to the tumor’s pattern. In this paper, we propose a new lightweight segmentation model called MicrowaveSegNet (MSegNet), which segments the brain tumor, and a new classifier called the BrainImageNet (BINet) model to classify the RMW images. Initially, three hundred (300) RMW brain image samples were obtained from our sensors-based microwave brain imaging (SMBI) system to create an original dataset. Then, image preprocessing and augmentation techniques were applied to make 6000 training images per fold for a 5-fold cross-validation. Later, the MSegNet and BINet were compared to state-of-the-art segmentation and classification models to verify their performance. The MSegNet has achieved an Intersection-over-Union (IoU) and Dice score of 86.92% and 93.10%, respectively, for tumor segmentation. The BINet has achieved an accuracy, precision, recall, F1-score, and specificity of 89.33%, 88.74%, 88.67%, 88.61%, and 94.33%, respectively, for three-class classification using raw RMW images, whereas it achieved 98.33%, 98.35%, 98.33%, 98.33%, and 99.17%, respectively, for segmented RMW images. Therefore, the proposed cascaded model can be used in the SMBI system.

## 1. Introduction

Nowadays, brain anomalies such as brain tumors are one of the serious causes of death worldwide. A brain tumor is the expansion of abnormal cells that are created inside the head. It causes harm to the brain’s major tissues and develops into cancer. Brain cancer can be fatal, crucially affect one’s quality of life, and it poses a threat to human life. Due to the uncontrolled growth of brain tumors, the possibility of developing brain cancer is increasing day by day. Brain tumor analysis, classification, and detection are severe issues for radiologists and medical doctors. The accurate and timely investigation of brain cancer is imperious for the appropriate treatment of this disease. Brain tumor segmentation can be a vital technique in medical imaging applications that segment the specific tumor regions from the head image. Additionally, the automatic segmentation of brain tumors from clinical images is important for the clinical assessment and planning of brain cancer treatments. According to the American Cancer Society, brain cancer is the 10th leading cause of death for adults and children [1]. However, the initial detection, classification, and proper investigation of brain tumors are particularly important to treat the tumor sufficiently. At present, different types of imaging technologies: PET (positron emission tomography), magnetic resonance imaging (MRI), ultrasound screening, X-ray screening, and CT (computed tomography) are utilized to diagnosis brain tumors in advanced healthcare facilities [2,3,4]. These imaging standards help physicians and radiologists identify different types of health-related diseases, such as brain cancer [4]. The crucial drawbacks of these imaging modalities are they increase the risk of a cancerous hazard because of their high dose radioactivity, lower susceptibility, high ionizing properties of brain tissues, expense, and risk for pregnant women and old patients [4,5,6,7,8,9,10]. Microwave imaging (MWI) showed excellent attention to the researchers for medical applications due to its great features such as its non-ionizing radioactivity, penetration capability with low power, non-invasive, risk-free ionization for the human body, and that it is cost-effective with a low profile [11,12,13]. Recently, researchers have used microwave imaging technology to overcome the drawbacks of the traditional medical imaging modalities [12,13,14,15,16,17,18,19,20]. Antenna plays an important role in microwave head imaging (MWHI) technology, where single-antenna sensors act as transmitters and others act as receivers. Receivers receive the backscattered bimedical signals, which are then processed by utilizing the image reconstruction algorithm. The image reconstruction algorithm is then applied to post-process the data to generate reconstructed images. Different image reconstruction algorithms have been used in microwave head imaging modalities to detect brain tumors [11,12,15,17,18,20,21,22,23,24,25]. However, the main limitations of the developed MWHI modalities are that they are (i) noisy, blurry, and the images created by the system are of a low resolution, (ii) the identification of the tumor with its location is complicated for a non-expert physician and radiologist, and (iii) there is difficulty in detecting tumor regions by automatic detection. To overcome such limitations, researchers have been applying deep learning techniques in microwave imaging systems [26,27,28,29,30,31].

Deep learning is a kind of machine learning modality that can use the convolutional neural network (CNN) model to classify and detect target objects. CNN has convolutional layers for feature extractions and densely connected layer(s) for classification. Recent advances in brain tumor segmentation have been made possible by deep learning methods such as CNNs [32]. On the other hand, image classification is the essential role of medical image analysis, in which deep convolutional neural networks (DCNNs) have been used for the last ten decades. The image classification identifies whether the target object or disease is present or not in the image of the investigation. Despite the fact that various deep neural network-based segmentation models have been proposed for brain tumor segmentation, nnU-net is the first segmentation model that is built to deal with the dataset diversity [33]. It optimizes and automates the crucial choices needed to create an effective segmentation pipeline for any given dataset. Additionally, a U-net is used in medical applications to segment brain tumors [34]. Thereafter, different modified versions of the U-net were used in image segmentation applications [35]. Based on U-net, the stacked multi-connection simple reducing net (SMCSRN) model was proposed for MRI brain tumor segmentation [36]. In this approach, the network is a combination of three U-net models created by applying 240 × 240 image datasets as an input. It takes a long time to train the model and fails to segment the small-sized tumor in the image due to the deeper architecture. A hybrid two-track U-net model was proposed in [37] to segment the brain tumor automatically. The architecture was trained and tested using the publicly available BRATS MRI dataset 2018 and received an 80% Dice score. It might be a problem to segment the tumor near the skull due to over downsampling. A multi-cascaded CNN model was proposed to segment the tumor in MRI images [38]. The architecture obtains multi-scale features by using a multi-cascade network to segment the tumor. The method used a coarse-to-fine segmentation framework to evaluate the public BRATS 2013–2018 datasets. However, the model was trained and tested on a small dataset and achieved a Dice score of up to 87%. A 3D Unet such as the S3D-Unet architecture was proposed to segment tumors in 3D images [39]. The maximum Dice score was only 78%, which means that a small area of the tumor cannot be segmented in the images. The training and testing accuracy were comparatively low for this network. In another study, a pre-trained DenseNet201 model was proposed to classify the tumors [40]. It is based on multilevel features and concatenation characteristics that can diagnose the tumor at an early stage. The approach achieved a 99.34% testing accuracy, but the precision score and Dice score were 92% and 83%, respectively.

The dual pathway Densnet architecture model was proposed in [40] to segment and classify tumor regions. The architecture was evaluated on the BRATS 2017 MRI dataset. The reported precision, F1 score, and Dice score were 85%, 88%, and 89%, respectively. The network model can only segment large areas of the tumor and not the small regions, resulting in comparatively poor classification performances. The deep ResNet FPN-based dilate network with middle supervision (RDM-Net) was used in [41] for segmenting the multimodal brain images. The network’s performance was evaluated using the BRATS 2015 dataset, and it achieved 86%, 71%, and 63% Dice scores in segmenting the complete tumor, core tumor, and enhanced tumor regions, respectively. The architecture fails to segment the small-sized tumors. In [42], a multi-scale CNN (MSCNN)-based tumor classification and image segmentation architecture was proposed. This network shows a better performance for specifying the tumor shape and location in the image. The model is heavy due to the deeper architecture; however, it cannot identify the small-shaped tumor location reliably. A multi-class tumor image classification by ResNet-50 was proposed in [43]. The model used a global average pooling mechanism to enhance the classification accuracy, but it achieved a 97.08% mean accuracy and a 90.02% F1 score.

Recently, operational neural networks (ONNs) have been applied as a diverse network standard for image analyzing, classification, and processing due to their non-linear properties, low computational complexity, simplicity in structure, and high performance. A self-organized ONN (Self-ONN) model was proposed in [44,45] to classify the biomedical images. It is seen that the Self-ONN model can perform better than conventional CNN models if the model architecture and parameters can be tweaked carefully. Since all the above-mentioned works used deeper architectures, it is natural that these networks require longer training and inference times and are not suitable for portable device deployment. Therefore, there is a demand to design a lightweight deep learning-based segmentation model to segment the tumor region from the reconstructed microwave (RMW) brain images, and also a lightweight classification model to classify the RMW brain images with a better classification performance. The main contributions of this work are specified below:

1. To the best of our knowledge, this is the first paper to propose a lightweight segmentation model called MicrowaveSegNet (MSegNet) that can automatically segment the desired brain tumors in RMW brain images from the sensors-based MBI system.
2. A lightweight classification model called BrainImageNet (BINet) is proposed to classify the raw and segmented RMW brain images using a new machine learning paradigm, the self-organized operational neural network (Self-ONN) architecture.
3. To segment both large and small brain tumors, the proposed MSegNet model is developed and tested on RMW brain tumor images.
4. We formulated a tissue-mimicking head phantom model to investigate the imaging system for generating the RMW brain image dataset.
5. A new Self-ONN model, BINet, three other Self-ONN models, and two conventional CNN classification models are investigated on the raw and segmented RMW brain tumor images to classify non-tumor, single tumor, and double tumor classes to show the efficacy of the proposed BINet classification model.

The rest of the article is organized as follows: Section 2 explains the experimental setup of a sensor-based microwave brain imaging system and the sample image collection process. The research methodology and materials, including the dataset preparation and experimental methods, are discussed in Section 3. Section 4 discusses the results of segmentation and classification models for the raw and segmented RMW images. A discussion point regarding classification classes is presented in Section 5. Finally, the paper is concluded in Section 6.

## 2. Experimental Setup of a Sensor-Based Microwave Brain Imaging System and Sample Image Collection Process

In this research, an experimental sensor-based microwave brain imaging (SMBI) system has been developed to generate microwave brain images and analyze the system’s performance. The SMBI system framework has been implemented by our research group, as reported in [23,46]. It is worth mentioning here that a wideband antenna sensor with a high gain and unidirectional characteristics is required with a frequency band of 1 to 4 GHz for the SMBI system [11,12,15,24,25].

### 2.1. Antenna Sensor Design and Measurement

**Figure 1:**
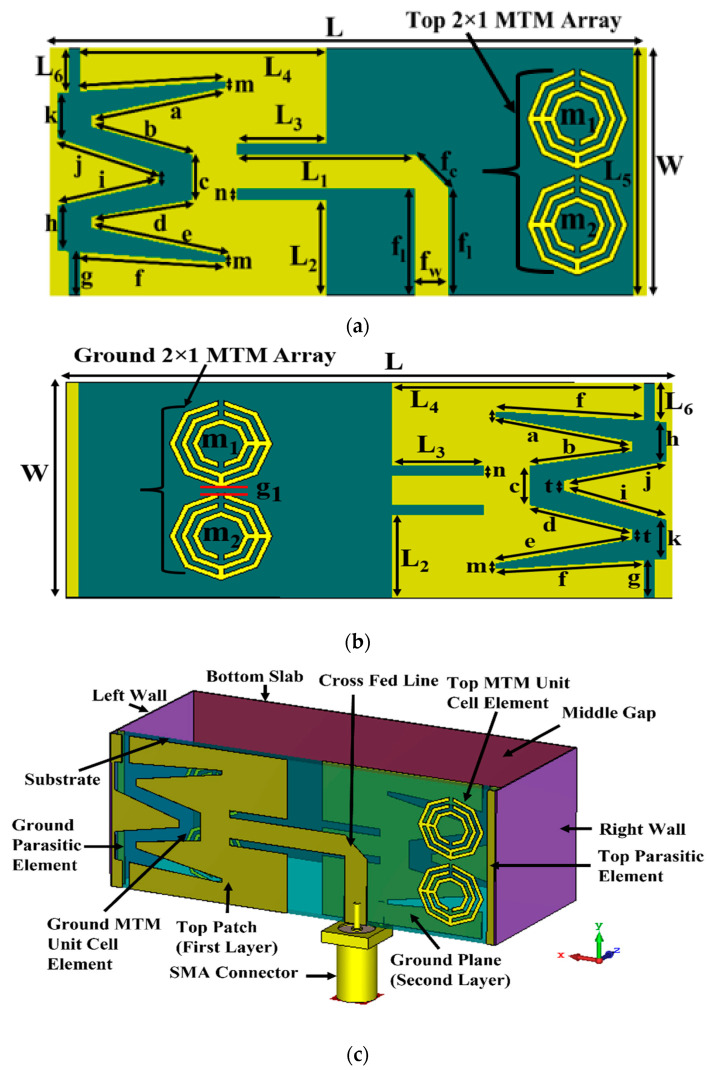
Graphic diagram of the 3D antenna sensor: (**a**) top view, (**b**) bottom view, (**c**) perspective view.

A new spider net-shaped triple split-ring resonator (SNTSRR) metamaterial (MTM) loaded three-dimensional (3D) wideband antenna sensor was constructed and printed on low-loss Rogers RT5880 material with a 0.0009 loss tangent, 2.20 relative permittivity, and 1.575 mm thickness, which ensures the requirements of producing the desired brain images. The schematic diagram of the MTM-loaded 3D antenna sensor structure is depicted in Figure 1. The geometric parameter values of the designed antenna sensor are presented in Table 1.

Initially, the plain patch is designed on the top side and back sides of the substrate. Then, an M-shaped slot and rectangular-shaped slots are cut out from the top side and back side to attain the required wideband frequency band. Walls of a twenty (20) mm length and twenty-two (22) mm width, with 0.2 mm thick copper, are attached at the left side and right side of the substrate towards the -z-direction. Thereafter, the fifty-three (53) mm long and twenty-two (20) mm wide bottom slab, of a 0.2 mm thickness, is attached to the left- and right-side walls to make it a 3D antenna.

The side walls (i.e., left wall and right wall) and bottom slab help to increase the gain and radiation directivity of the antenna. The sensor is fed at the top layer with a 50 Ω cross-fed line via an SMA connector. Then, a 2 × 1 MTM array structure is placed on the top and the bottom sides of the antenna sensor for enhancing the antenna sensor’s gain, efficiency, and radiation directivity. The antenna sensor is designed and simulated by the computer simulation technology (CST) simulator software. The optimized antenna dimension is 53 × 22 × 21.575 mm^3^ (i.e., length (L) × width (W) × height (H), where H = middle gap (h = 20 mm) + substrate thickness (Th = 1.575 mm).

**Figure 2:**
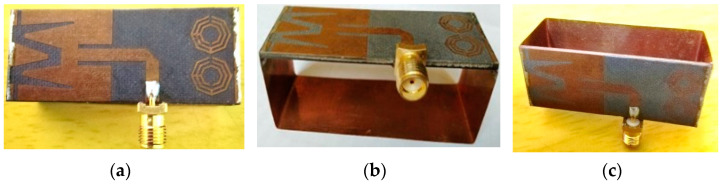
Fabricated prototype of the SNTSRR MTM-loaded 3D antenna: (**a**) top view, (**b**) side view, (**c**) perspective view.

**Figure 3:**
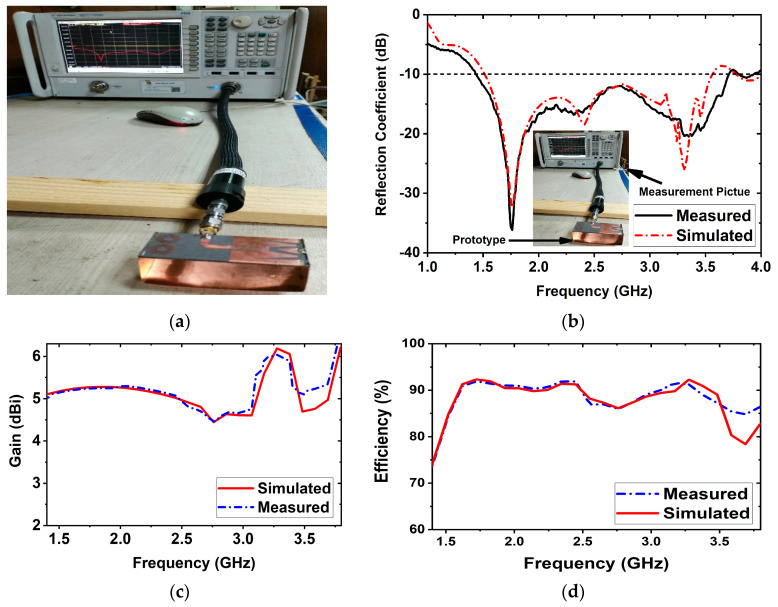
Measurement setup and resultant outcomes of the antenna sensor: (**a**) PNA setup, (**b**) measured and simulated reflection coefficient, (**c**) gain, (**d**) efficiency.

Figure 2 illustrates different views of the fabricated prototype of the antenna sensor. The fabricated sensor is set up with the PNA and then measured for the scattering parameters (i.e., reflection coefficient). The antenna measurement pictures are illustrated in Figure 3a. The measurement is performed within the frequency range of 1 GHz to 4 GHz. The simulated and measured scattering parameters |S11| (i.e., reflection coefficient) are illustrated in Figure 3b.

It was discovered, depicted in Figure 3b, that the measured frequency band of the sensor is 1.43 GHz to 3.71 GHz, with a maximum resonance of −37 dB at 1.76 GHz, whereas the simulated operating frequency of the antenna is 1.51 GHz to 3.55 GHz with a maximum resonance of −32 dB at 1.76 GHz. The attained operating frequency band of 1.43 GHz to 3.71 GHz is used for microwave brain imaging as a compromise between the signal penetration in the head tissues and the image resolutions. Except for a slight shift in the resonances to the lower frequencies caused by fabrication or soldering tolerance, both measured and simulated results show a good agreement. The antenna sensor’s measured gain is 6.03 dBi with a maximum efficiency of 91%, as shown in Figure 3c,d.

### 2.2. Phantom’s Composition Process and RMW Image Sample Collection

**Figure 4:**
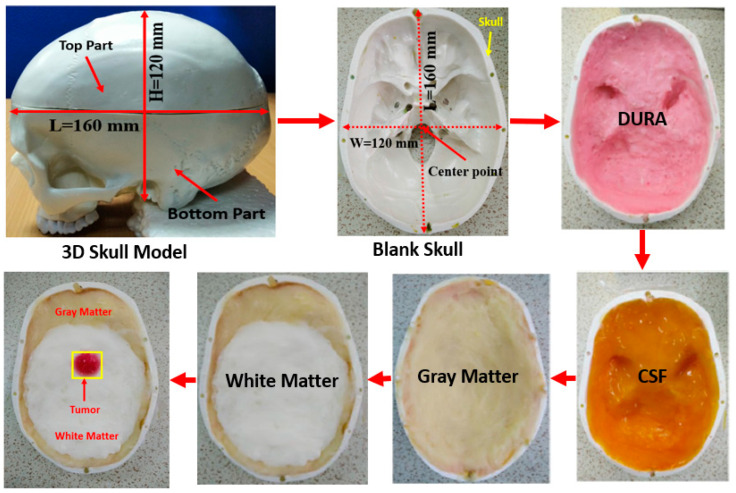
Phantom’s composition process using fabricated four tissues.

A four-layered (i.e., DURA, CSF, gray matter (GM), and white matter (WM)) tissue-mimicking brain phantom model is constructed and utilized for evaluating the performance of the system in this study. The tissue layers and tumors were fabricated according to the recipe described in [47]. The length and height of the 3D skull are L = 160 mm and H = 120 mm, respectively. However, at first, DURA was fabricated and filled into a 3D human skull, then CSF, white matter, and gray matter were filled step by step in the model. After that, the fabricated tumor(s) were placed in different locations for image reconstruction purposes. The phantom’s composition steps are illustrated in Figure 4.

#### RMW Brain Tumor Image Sample Collection

**Figure 5:**
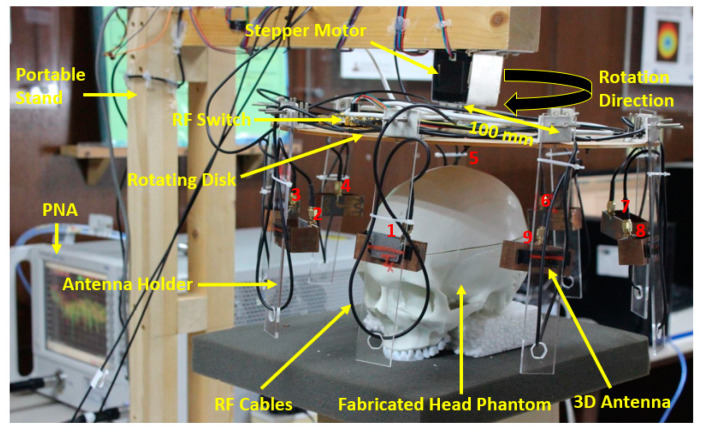
Experimental setup for sensors-based microwave brain imaging system [46].

In this research, we utilized our new 3D antenna sensor in the implemented SMBI system framework [46] to generate microwave brain images. The experimental SMBI system is illustrated in Figure 5. The system has a circular-shaped rotating disk with a nine-antenna sensor array holder for holding the antennas. The mounting framework is connected to a portable stand via a stepper motor, rotating from 0 to 360 degrees. A fabricated four-layered phantom model, including the tumors, has been placed in the center of the framework. In addition, the tumors were inserted into several locations on the phantom to generate RMW brain tumor images. The mounted nine-antenna sensor array framework is rotated around the head model through the stepper motor, where one antenna sensor transmits the microwave signals towards the head phantom, and then backscattered signals are received by the remaining eight antenna sensors. The received backscattered bio signals (S21, S31, S41, ……, S91) were collected in each 7.2° degrees rotation and measured by the PNA (power network analyzer). Therefore, a total of 9 × 8 × 50 locations were scanned around the phantom via the system to investigate its performance.

**Figure 6:**
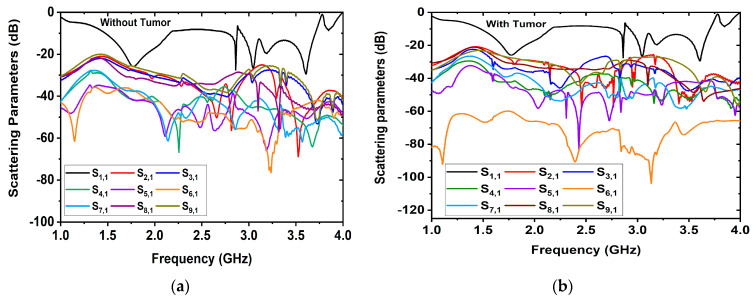
Reflected scattering bio signals received by the receiving antenna sensors: (**a**) without tumor, (**b**) with tumor.

For image reconstruction purposes, we considered two scenarios: a tissue-mimicking head phantom without tumors and with tumors. The reflected biosignals received by the antenna sensors are presented in Figure 6. The signals were collected by the PNA. Figure 6a illustrates the reflected signals when a tumor was not present in the head model, and Figure 6b illustrates the reflected signals, when the tumor was present in the head model. The image processing unit is directly connected to the PNA. The collected signals from the PNA were preprocessed by the MATLAB programming language. Thereafter, an image reconstruction algorithm, M-DMAS (modified delay-multiply-and-sum) [23] was utilized to produce the desired RMW brain images of the head regions. The frequency range of 1.43 GHz to 3.71 was considered for image reconstruction. After that, the produced images were sampled by the Origin pro data analyzer software to set the axis with respect to the brain regions. These processes were repeated by the program and collected a set of RMW image samples for further processing. The used imaging algorithm can reconstruct only two tumor-based images and detect a minimum of a 5 mm small-sized tumor. The minimum separation (i.e., resolution) for the algorithm to distinguish two tumors from each other is approximately 38 × 38 pixels (i.e., a minimum 10 mm distance between two tumors, where 1 pixel = 0.2645833333 mm).

**Figure 7:**
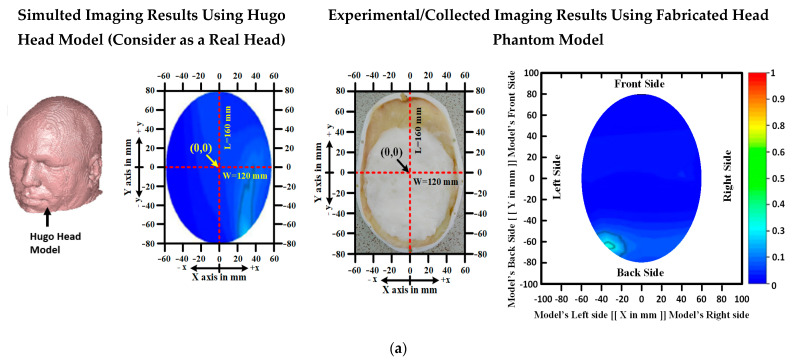

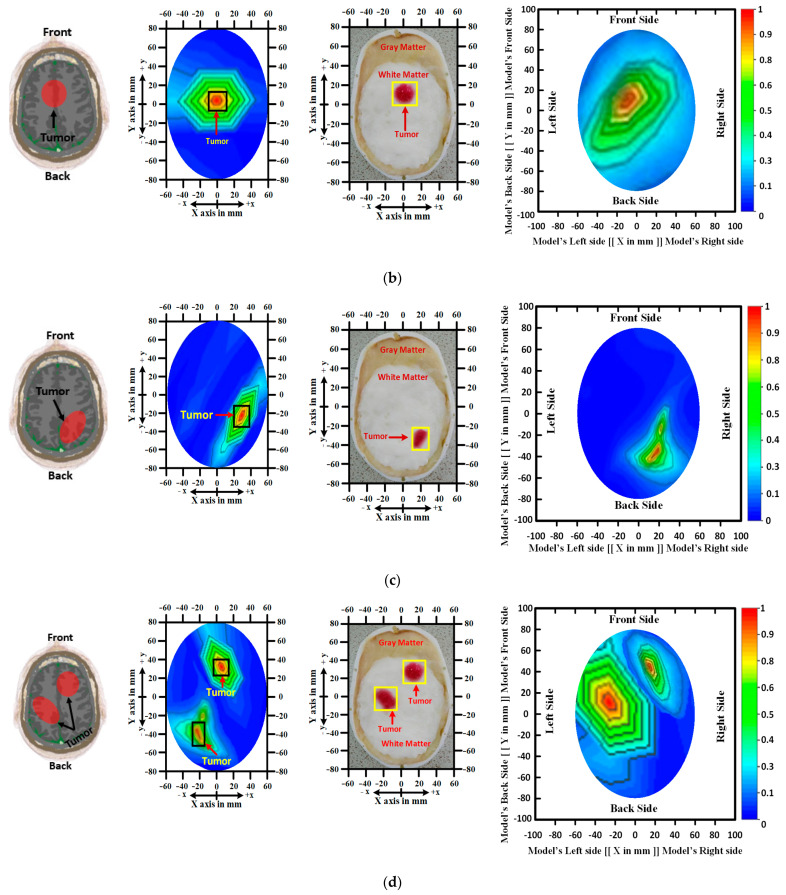
Comparision imaging results with simulated as considering real situations and formulated tissue−imitating phantom models with reconstructed images: (**a**) non-tumor, (**b**,**c**) single tumor, (**d**) double tumors.

However, in practice, we need to compare the RMW images with the real head for verifying the image reconstruction accuracy, so that the system can be used in real situations (i.e., clinic or hospital); despite this, due to clinical permission issues in the lab, we cannot use live human heads for comparing the imaging outcomes of the fabricated phantom model. However, we compared our imaging outcomes with a simulated “Hugo Head” model, which acts as a real head [48]. The Hugo model ensures the dielectric properties of real brain tissues. We placed the tumor(s) in different locations on the Hugo model and compared the image samples with a fabricated head model. The cartesian coordinate system can be applied to detect the location of the tumor in the images. The simulated (considering real situations) and experimental/collected RMW brain image samples with their coordinates are illustrated in Figure 7. It is observed from Figure 7 that the RMW images with tumor locations are almost the same and show a good agreement. In addition, for verifying the imaging results, the comparison results with the other imaging systems and reconstruction algorithms are presented in Table 2. It can be seen from Table 2 that the used imaging system and algorithm performed better than other imaging systems and algorithms.

Furthermore, the tumors were placed in different locations on the model, and 300 samples were collected, including non-tumor, single tumor, and double tumor cases, to create the dataset. Later, image preprocessing and augmentation methods were applied to the collected image sample dataset to produce a large enough training and testing dataset. Due to the lack of image diversity, the proposed models were trained and tested using the same phantoms. However, it is possible to test the models by using different phantoms, which is our future work. The proposed segmentation model was utilized for segmenting the tumor regions, and a classifier model was investigated on the raw and segmented RMW brain images. Two experiments were carried out with the training dataset to segment the tumor(s) and then classify the RMW brain images.

## 3. Methodology and Materials

**Figure 8:**
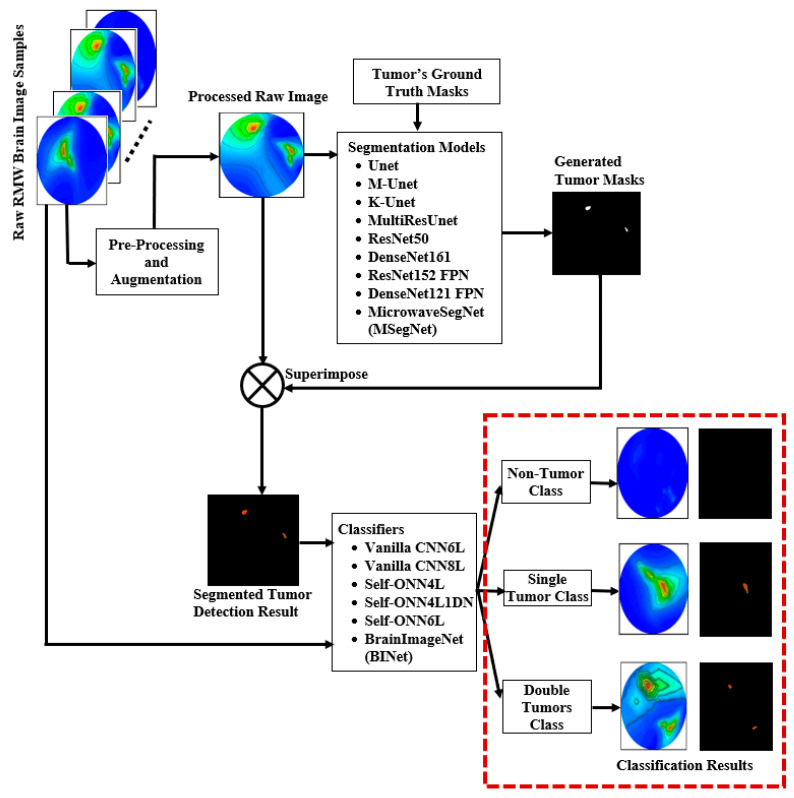
The complete methodology flow chart of the research work.

The study’s overall methodology is covered in this section, along with the dataset description, pre-processing, data augmentation methods, and experimental analysis. The comprehensive methodology of the research work is presented in Figure 8. This research utilized RMW brain images, which were obtained from the implemented experimental brain imaging system, as reported in our previous work [46]. The brain images, including non-tumor, tumor, and corresponding segmented tumor region masks, are obtained. As previously indicated, the study primarily uses two types of images: (i) healthy brain images (i.e., non-tumor images) and (ii) unhealthy brain images (i.e., tumor-based images). The unhealthy images are classified into two categories: (i) single tumor images and (ii) double tumor images.

The work first explored the proposed lightweight MSegNet segmentation model along with nine other state-of-the-art segmentation models to investigate the segmentation performance of detecting tumor regions in the RMW brain images. At first, raw RMW brain image samples were collected, and then image pre-processing was applied. In addition, the corresponding ground truth masks are also created and then applied along with the image dataset. Thereafter, a tumor mask is superimposed on the raw RMW images to create a segmented tumor region-based image dataset. Then, the proposed lightweight BrainImageNet (BINet) classification model and five other CNN-based classification models were used to investigate the classification performances of the raw and segmented RMW brain images for three class classifications: non-tumor, single tumor, and double tumor. The details of the sub-sections are discussed below.

### 3.1. Dataset Preparation

**Figure 9:**
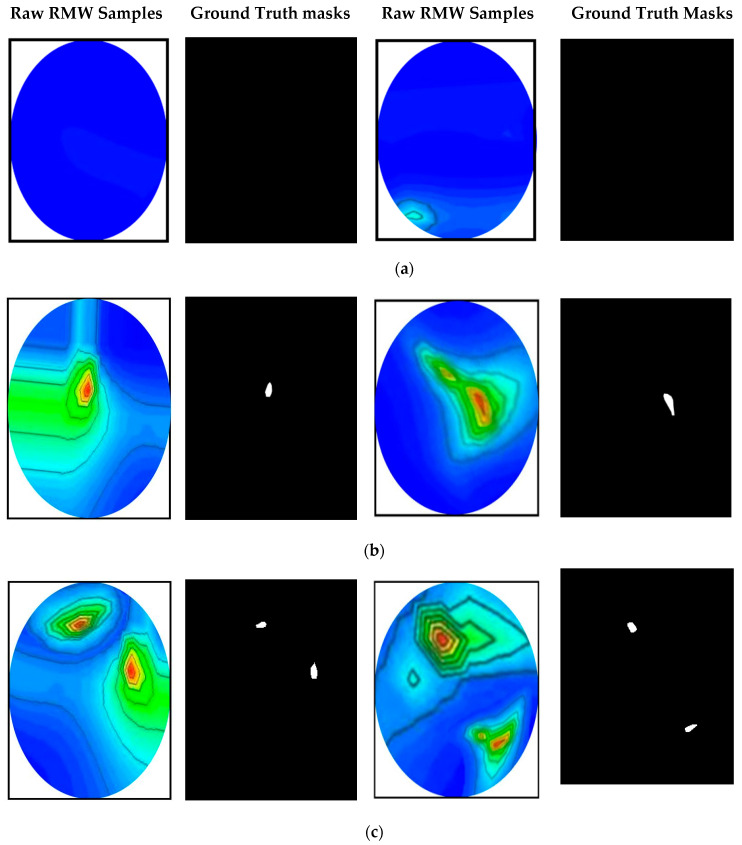
The RMW brain image samples and their corresponding ground truth masks from the original dataset: (**a**) non-tumor, (**b**) single tumor, (**c**) double tumors.

The RMW brain images and their corresponding ground truth masks are used as an original dataset in this research work. The original dataset consists of 300 RMW images, where one hundred images are in the non-tumor (i.e., healthy brain) class and two-hundred images are in the tumor (i.e., unhealthy brain) class, and corresponding ground truth masks are made available in the dataset. The tumor class is further divided into two subclasses: 100 images for a single tumor and another 100 for double tumors. Samples of the raw and segmented RMW brain images and their ground truth masks of the dataset are shown in Figure 9.

### 3.2. Image Pre-Processing and Method of Augmentation

This section goes over image processing and data preparation for deep learning techniques. The pre-processing method is the initial step of a DL (deep learning) model due to its input constraints. The different CNN network models, including the segmentation and classification models, have different input size requirements. Thus, images are pre-processed (resized and normalized) before training the models. The images are resized to 256 × 256 pixels for the investigation of ten Unet segmentation network models such as: (i) U-net, (ii) Modified Unet (M-Unet), (iii) MultiResUnet, (iv) Keras Unet (K-Unet), (v) Unet with ResNet50 backbone, (vi) Unet with DenseNet161 backbone, (vii) ResNet152 FPN, (viii) DenseNet121 FPN, (ix) nnU-net, and (x) proposed MSegNet. On the other hand, for the raw and segmented brain tumor image classification purposes, the images are resized to 224 × 224 pixels for a vanilla CNN, three Self-ONNs, and the proposed BINet models. Using the mean (M) and standard deviation (STD) of all images in the original dataset, the z-score normalization method is used to normalize the images. Deep learning models typically require a large image dataset to effectively train a model to segment and classify the target object regions in the image.

**Figure 10:**
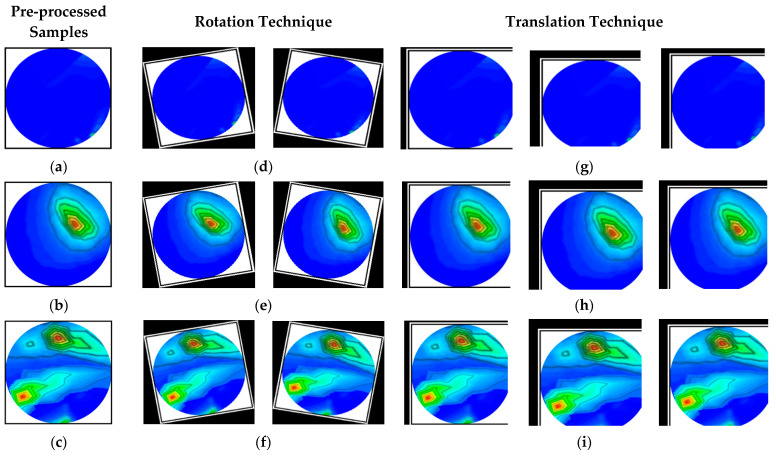
Augmented sample of training set: (**a**–**c**) pre-processed non-tumor, single tumor, and double tumor images, (**d**–**f**) images after rotation by 20 degrees counterclockwise and clockwise for non-tumor, single tumor, and double tumors, (**g**–**i**) images after three percent horizontal, five percent vertical and horizontal, and five percent horizontal and three percent vertical translation for non-tumor, single tumor, and double tumors.

In this study, the image augmentation technique is employed to create a large training dataset for the deep segmentation models because our tiny dataset is unsuitable for training them. In this research, three different image augmentation strategies (e.g., rotation, scaling, and translation) are utilized to generate the training image set. The images are rotated in both clockwise and counterclockwise directions at an angle ranging from 3 to 50 degrees. The tumor objects are thus relocated at various locations within the images. Scaling is the process of reducing or enlarging the size of an image. In this case, image magnifications range from 2% to 15%. The image translation technique shifts the tumor objects to different locations in the images by translating the images by 3–10% vertically and horizontally. After pre-processing and augmentation, samples of the augmented images are illustrated in Figure 10.

### 3.3. Dataset Splitting and Ratio Consideration for Training and Testing Dataset

Dataset splitting is a technique for evaluating the performance of a deep learning model. It is not good practice to use the entire dataset for training the model because if the entire dataset is used to train the model, we will not be able to assess the performance of the proposed model and an overfitting problem may occur. For that reason, proper dataset splitting is essential for the model. Typically, the dataset is split into three sets: the training, testing, and validation sets, with 60% for the training, 20% for the testing, and 20% for the validation, but the exact ratio depends on the collected dataset and model architecture. In this work, the original image dataset was split into three sets, the training, testing, and validation sets, and the appropriate percentage was set for all splits by considering the model’s architecture and the small dataset as well as using the K-fold cross-validation technique to avoid overfitting. Thus, this study uses a five-fold cross-validation technique for training, validation, and testing purposes. Additionally, a random shuffling method was applied to the dataset before making three splits so that every split had an accurate representation of the dataset. Based on the architecture and image dataset, 80% of the total images were utilized for training, and 20% were used for testing in order to do a five-fold cross-validation. Additionally, 20% of the training dataset, which comprises 80% of the dataset, is used for validation to prevent overfitting. Thus, the performances were measured on five-fold cross-validation data which indicates more generalized performance. After augmentation, 6000 images were created per fold for training the model. Table 3 displays a thorough overview of the image dataset.

### 3.4. Experiments

In this study, two sets of experiments (brain tumor segmentation, classification with raw and segmented RMW brain images) were carried out. All segmentation models in this work are implemented using the PyTorch library and Python 3.7 on the Anaconda distribution platform. The experiments are run on a 64-bit version of Windows 10 with 128 GB of RAM and a 3.30 GHz 64-bit Intel(R) Xeon(R)W-2016 CPU. A 32 GB NVIDIA GeForce GTX 1080Ti GPU is also utilized to speed up network training operations. The two sets of experimental analysis (brain tumor segmentation and RMW image classification) are explained in the following sections. Finally, the average of the performance metrics of the five folds was calculated.

#### 3.4.1. Proposed MicrowaveSegNet (MSegNet)—Brain Tumor Segmentation Model

**Figure 11:**
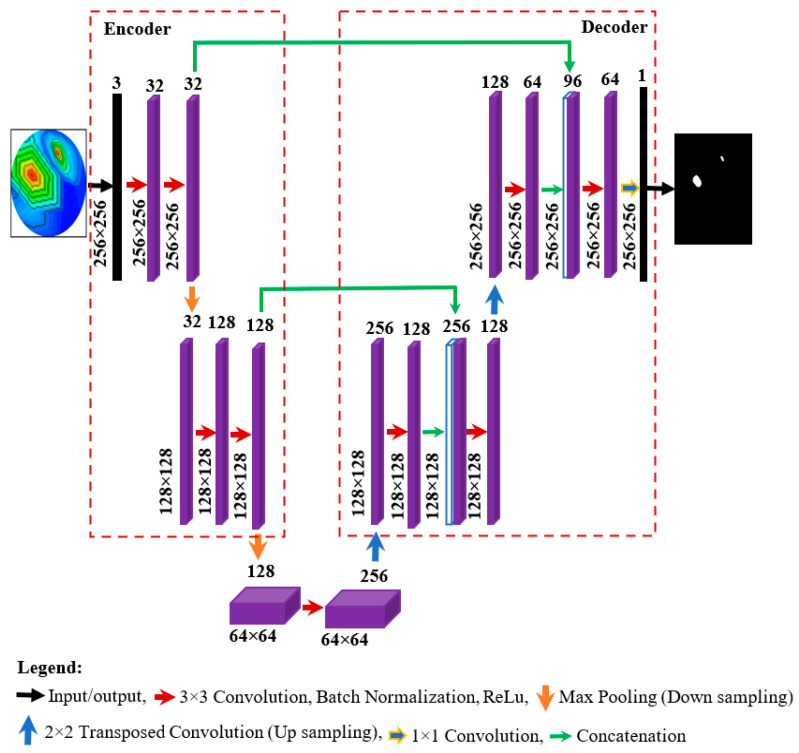
Proposed lightweight MicrowaveSegNet (MSegNet) model for tumor segmentation.

Brain tumor segmentation is done to segment the tumors from the RMW brain images to identify the correct spatial location of a tumor in the images. Nowadays, U-net-based deep learning architecture is popularly used to segment objects in medical imaging applications [55]. The main benefit of this network is that it can precisely segment the target features and effectively process and evaluate the images [36,56]. This study proposed a lightweight segmentation model, called MicrowaveSegNet (MSegNet). The proposed MSegNet model architecture is illustrated in Figure 11. Typically, a U-net model has four encoding and decoding blocks and some skip connections. The MSegNet model used only two levels in both encoding and decoding to make it a lightweight network. The model consists of a contracting path with two encoding blocks followed by an expanding path with two decoding blocks.

Each encoder and decoder block is made up of two 3 × 3 convolutional layers, followed by a non-linear activation function. The input image (256 × 256) is fed into the encoder of the network. Each encoding block is made up of two 33 convolutional layers followed by a 2 × 2 max-pooling layer for down sampling. Every decoding block in the decoder consists of an up-sampling followed by one 3 × 3 convolutional layer, a concatenation layer, and another 33 convolutional layers. For up-sampling, the decoder starts with a 2 × 2 transposed convolutional layer. All convolutional layers in both the encoder and decoder are followed by the BN (batch normalization) and rectified linear unit (ReLu) activation functions. The contracting path from the encoder block is directly connected with the decoder block’s concatenation layer to create a high-resolution segmentation feature map. At the ending layer, 1 × 1 convolution is used to create the output map from the last decoding block to two-channel feature maps. Thereafter, the Softmax function is utilized in two-channel feature maps to map every pixel into a binary class of background or tumor.

#### 3.4.2. Experimental Analysis of the Segmentation Models

For the experimental purposes, the proposed MSegNet model and other eight models (as mentioned earlier) were trained and validated by using a five-fold cross-validation image dataset to evaluate the tumor segmentation performance. The training was executed using a learning rate (LR) of 0.0005 for a maximum of 30 epochs, batch size of 8, and utilized Adam optimizer for network optimization. During training, if no improvement was observed for ten successive epochs, then the learning rate was decreased by a learning factor of 0.2 and the training is stopped if there was no improvement detected for 15 successive epochs. The complete hyperparameters for all the models are shown in Table 4.

**Figure 12:**
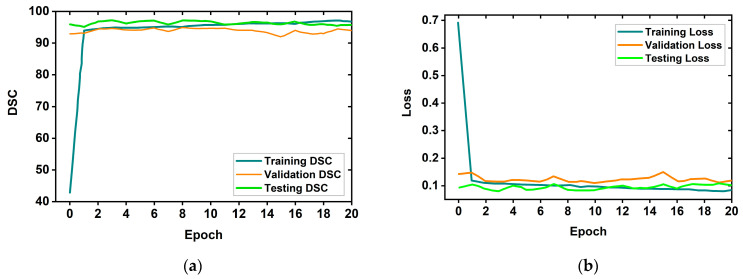
The training results graph: (**a**) the DSC graph, (**b**) loss graph.

Moreover, the Dice score (DSC) and loss plots for different epochs during the training of the proposed MSegNet model is presented in Figure 12. As can be observed from Figure 12, the model was trained for 20 epochs and the model’s performance became saturated after a few epochs in terms of the DSC and loss. So, it can be seen that the proposed model is not over-fitting and converges well and should segment the desired tumor regions in the RMW brain images reliably.

#### 3.4.3. Proposed BrainImageNet (BINet)—Brain Image Classification Model

Recently, an operational neural network (ONN)-based model was introduced in [57] to overcome the linear nature of the CNN. The ONN is a diverse network that has demonstrated a promising performance in a number of applications, including image denoising and image restoration. It usages a permanent set of non-linear operators to discover complicated patterns from any input [58,59]. On the other hand, the fixed set of operator libraries restricts ONNs ability to learn. To overwhelm this issue, self-organized ONN (Self-ONN) is offered in [60]. Instead of using a static group of operator libraries, Self-ONN unavoidably discovers the best set of operators over the course of training. As a result, the model becomes more solid, able to handle a wider range of situations, and capable of making accurate generalizations. Self-ONN networks choose the best set of operators during the training process, which can be a combination of any standard function or some other functions that we do not know. The output $O_{k}^{L}$ at kth neuron of Lth layer of any ONN can be illuminated as follows [45]:

(1) $$O_{k}^{L}=b_{k}^{L}+\sum_{i=1}^{N_{L-1}}\Psi_{ki}^{L}\left(w_{ki}^{L},y_{i}^{L-1}\right)$$

where $b_{k}^{L}$ and $w_{ki}^{L}$ are the biases and weights related to the neuron and layer, $y_{i}^{L-1}$ is the input from the preceding layer, $N_{L-1}$ is the kernel size, and $\Psi_{ki}^{L}$ is the nodal operator of the neuron. If $\Psi_{ki}^{L}$ is linear, then the equation simply corresponds to a conventional CNN. In ONN, the aggregate nodal operator Ψ can be formulated using a set of standard functions as follows [57]:

(2) $$\Psi \left(w,y\right)=w_{1}\sin(w_{2}y)+w_{3}\exp(w_{4}y)+\ldots \ldots +w_{q}y$$

Here, w denotes the *q*-dimensional array of the parameters, which is composed of the internal parameters of the individual functions and weights. Instead of a static set of operators, the combined nodal operator Ψ can be formulated by utilizing a Taylor series function. The Taylor series function $f\left(x\right),$ near point, and x=a is stated by the following equation [57]:

(3) $$f\left(x\right)=f\left(a\right)+\frac{f^{\prime }\left(a\right)}{1!}\left(x-a\right)+\frac{f^{\prime \prime }\left(a\right)}{2!}{\left(x-a\right)}^{2}+\frac{f^{\prime \prime \prime }\left(a\right)}{3!}{\left(x-a\right)}^{3}+\ldots \ldots +\frac{f^{n}\left(a\right)}{n!}{\left(x-a\right)}^{n}$$

Equation (3) can be used to construct the nodal operator as follows:

(4) $$\Psi \left(w,y\right)=w_{0}+w_{1}\left(y-a\right)+w_{2}{\left(y-a\right)}^{2}+\ldots \ldots +w_{q}{\left(y-a\right)}^{q}$$

Here, $w_{q}=\frac{f^{\left(n\right)}\left(a\right)}{q!}$ denotes the qth parameter of the qth order polynomial. In Self-ONN, *tanh* has been employed as an activation function which is constrained at the range of [−1, 1]. So, for *tanh,* a is equal to zero in Equation (4).

**Figure 13:**
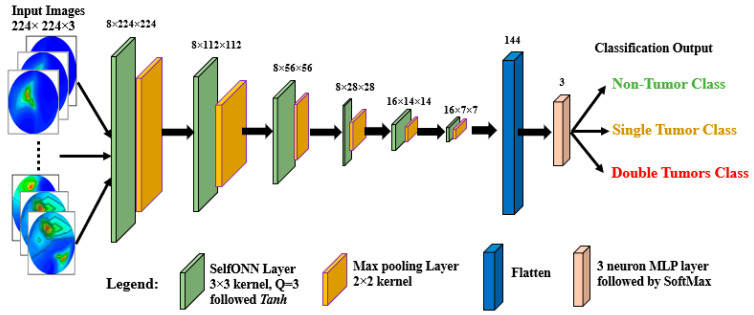
Proposed BrainImageNet using Self-ONN.

In this study, we developed a new lightweight classification model called BrainImageNet (BINet) to classify the raw and segmented brain tumor images. BINet is designed using a self-organized operational neural network (Self-ONN) architecture. The detailed architecture of the BINet classification model is shown in Figure 13. As illustrated in Figure 8, the BINet has six Self-ONN layers, where the first 4 layers have 8 neurons and the other 2 have 16 neurons, respectively. Through the self-organization of its nodal operators, it can accomplish the requisite non-linear transformations to extract optimal features from the brain tumor images. The kernel sizes are set as 3 × 3 for the Self-ONN layer and 2 × 2 for the max-pooling layer, respectively. Moreover, the Q value is set to 3 as the order of qth order polynomial for all operational layers. The input image of dimension 224 × 224 is fed to the input layer. The images are propagated through the Self-ONN and max polling layers and features are extracted into different feature maps. A flattening layer with 144 neurons is used to convert the output of the convolutional layer into a one-dimensional feature vector and apply it to the final dense layer. The network’s final classifier is the dense layer, which employs a three-neuron MLP layer followed by a SoftMax activation function to classify the upcoming images as non-tumor, single tumor, or double tumor.

#### 3.4.4. Experimental Analysis of the Classification Models

In this section, we discuss two classification experiments to investigate the classification performances of the networks: (i) classification using the raw RMW images (non-segmented) and (ii) classification using the segmented RMW images. However, the proposed BINet model and three variations of the Self-ONN-based model, such as 2 Self-ONN models with 4 operational layers and 1 with 6 operational layers (Self-ONN4L, Self-ONN4L1DN, and Self-ONN6L), as well as 2 vanilla CNN models with 6 and 8 layers (Vanilla CNN6L and Vanilla CNN8L), were investigated and the results were compared separately by using the raw (non-segmented) and segmented RMW tumor images. In the model names, “4L” means the model consists of four layers, “6L” means the model consists of six layers, and “1DN” means the model consists of one dense layer in the final stage. The training was executed using a learning rate (LR) of 0.0005 for a maximum of 30 epochs, batch size of 16, utilized Adam optimizer for network optimization, and set stop criteria based on the training loss. The Q order value is a significant factor during training the models; Q = 1 is set to train the two vanilla CNNs, and Q = 3 is set for the Self-ONN and BINet models. The hyperparameters for the classification models are presented in Table 5.

### 3.5. Performance Evaluation Matrices

#### 3.5.1. Assessment Matrix for the Segmentation Model

After completion of the training and validation phase, the tumor segmentation performances of the different networks (e.g., MSegNet and other eight network models) for testing the RMW brain image dataset are evaluated. The performance evaluation matrices are the accuracy (A), Intersection-over-Union (IoU), and Dice score (DSC), and these are calculated by the following equations [61]:

(5) $$A=\frac{\left(N_{TP}+N_{TN}\right)}{\left(N_{TP}+N_{FN}\right)+\left(N_{FP}+N_{TN}\right)}$$

(6) $$IoU=\frac{N_{TP}}{\left(N_{TP}+N_{FN}+N_{FP}\right)}$$

(7) $$DiceScore\left(DSC\right)=\frac{\left(2\times N_{TP}\right)}{\left(2\times N_{TP}+N_{FN}+N_{FP}\right)}$$

#### 3.5.2. Assessment Matrix for the Classification Model

The classification performance of the various CNN and Self-ONN models is evaluated by the five evaluation matrices, such as: (i) the overall accuracy (A), (ii) weighted recall or sensitivity (R), (iii) weighted specificity (S), (iv) weighted precision (P), and (v) weighted F1-score (Fs). The assessment metrics are computed by utilizing the following formulas [61]:

(8) $$A=\frac{\left(N_{TP}+N_{TN}\right)}{\left(N_{TP}+N_{FN}\right)+\left(N_{FP}+N_{TN}\right)}$$

(9) $$R=\frac{N_{TP}}{\left(N_{TP}+N_{FN}\right)}$$

(10) $$S=\frac{N_{TN}}{\left(N_{FP}+N_{TN}\right)}$$

(11) $$P=\frac{N_{TP}}{\left(N_{TP}+N_{FP}\right)}$$

(12) $$F_{s}=\frac{\left(2\times N_{TP}\right)}{\left(2\times N_{TP}+N_{FN}+N_{FP}\right)}$$

where *N_TP_* denotes the number of tumor images which were detected as tumors, *N_TN_* represents the number of non-tumor images which were detected as non-tumors, *N_FP_* denotes the number of images incorrectly identified as a tumor, and *N_FN_* denotes the number of images with tumor(s) that were missed by the network.

## 4. Results and Discussion

### 4.1. Brain Tumor Segmentation Performances

**Figure 14:**
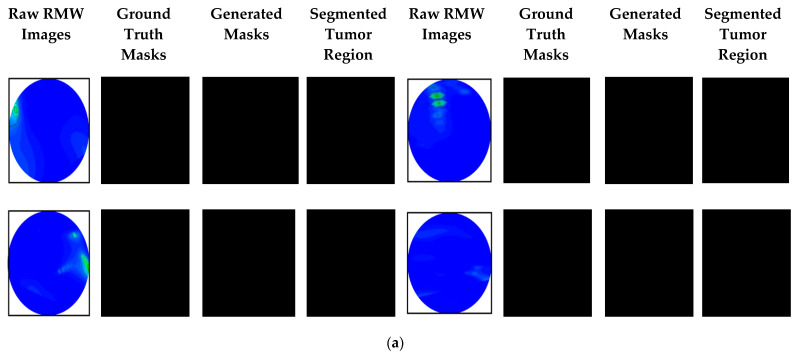

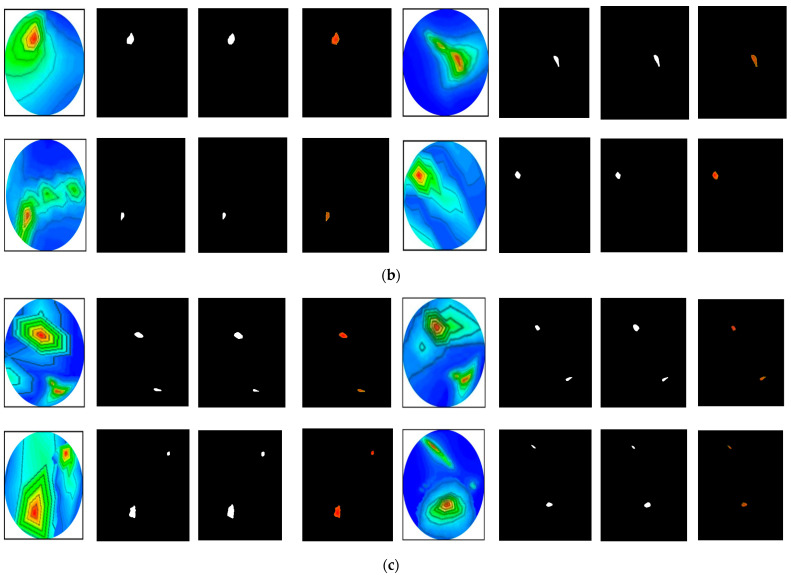
Proposed MSegNet model’s tumor segmentation results with ground truth masks, generated masks, and resultant segmented tumor images for: (**a**) non-tumor class, (**b**) single tumor class, (**c**) double tumors class.

It is notable that the main advantages of the MSegNet model are: (i) a lightweight architecture with only two layers in encoding and decoding blocks, (ii) a low training and inference time, (iii) it can segment the desired tumor (small and large) regions precisely with a high-resolution image, and (iv) it shows high segmentation performances in terms of the accuracy, IoU, and Dice score compared to other deeper segmentation networks. For experiment purposes, the proposed MSegNet model and other nine segmentation models (as mentioned earlier) were used to investigate the tumor segmentation performances. The tumor segmentation performance results of the MSegNet model are shown in Figure 14, which illustrates the non-tumor, single tumor, and double tumors images, corresponding to ground truth masks, generated masks, and the resultant segmented tumor regions of the raw RMW brain images.

It is observed that the MSegNet model precisely segmented the desired region of the tumor as an anomaly in the RMW brain images. The four evaluation performance matrices of the segmentation models are presented in Table 6. It is observed from Table 5 that the MSegNet model exhibited better performances compared to the other nine segmentation models. The achieved accuracy (A), IoU, Dice score (DSC), and loss of the proposed model are 99.97%, 86.92%, 93.10%, and 0.101, respectively. However, the high accuracy, Dice score, and low loss ensure that the MSegNet model can clearly segment the desired tumor regions in the raw RMW images. In addition, the computational complexity in terms of the parameter (M), training time (time taken to train the model) per fold, and inference time (time taken by the network model to segment tumor regions an input image) of the MSegNet model was compared with nine Unet-based segmentation models, presented in Table 7. The inference time per image was computed over the 48 images of the validation set. It can be observed from Table 7 that the MSegNet model has only eight network parameters and a low training and inference time that ensures the lightweight characteristics of the model.

### 4.2. Raw and Segmented RMW Brain Images Classification Performances

In this section, we discuss the three Self-ONNs (Self-ONN4L, Self-ONN4L1DN, and Self-ONN6L), two vanilla CNNs (vanilla CNN6L and vanilla CNN8L), and proposed BINet classification models to investigate the classification effectiveness by applying the raw and segmented RMW brain images. The classification models are able to classify the images into non-tumor, single tumor, and double tumors classes. The main advantages of the BINet model in this research are: (i) a lightweight architecture that uses a non-linear operation to boost the network diversity along with the classification effectiveness, (ii) the ability to optimize the learning weight of each layer during the training process, and (iii) that it attains superior classification performances while significantly reducing the computational complexity rather than conventional CNNs models.

All classification models were trained by using the raw RMW brain tumor images. The comparative statistical classification performance (with mean, standard deviation (STD) and paired *t*-test/*p*-value outcomes of the models for the raw RMW brain tumor images are presented in Table 8. It was investigated that the conventional deeper CNN networks have achieved lower performances than the three Self-ONNs models, but the BINet model was the best model among all the networks and achieved the highest performances. The BINet has exhibited a mean accuracy, precision, recall, specificity, and F1 score of 89%, 88.74%, 88.67%, 94.33%, and 88.61%, respectively, for the raw RMW brain images. Moreover, an STD accuracy, precision, recall, specificity, and F1 score of 3.49%, 3.58%,3.59%, 2.62%, and 3.59%, respectively, were obtained for the raw RMW brain images.

Then, we investigated the statistical classification performances of all mentioned classification models for the segmented RMW images. All models were trained by utilizing the resultant segmented RMW brain tumor images to verify the classification efficacy. The comparative statistical classification performances (with mean, standard deviation (STD), and paired *t*-test/*p*-value) of the models for classifying the segmented RMW brain tumor images into the three classes are presented in Table 9. It was observed that the conventional deeper CNN networks and Self-ONN models improved the performances but, the performances were lower than the BINet model. However, the BINet model was the best among all networks and attained the highest performances. The attained mean accuracy, precision, recall, specificity, and F1 score of the BINet model are 98.33%, 98.35%, 98.33%, 99.17%, and 98.33%, respectively. Furthermore, the STD accuracy, precision, recall, specificity, and F1 score of the BINet model are 1.45%, 1.44%, 1.45%, 1.03%, and 1.45%, respectively, for segmented RMW images. Therefore, it is concluded that the proposed classification model exhibited a better performance for the segmented RMW brain images.

### 4.3. Performance Analysis

**Figure 15:**
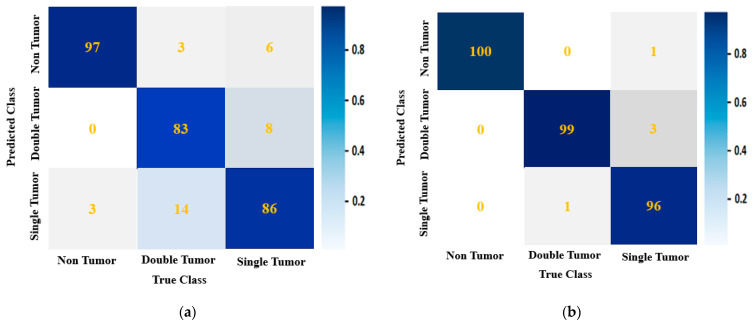
The confusion matrix of the proposed BINet classification model for: (**a**) the raw RMW brain images, (**b**) the segmented RMW brain images.

**Figure 16:**
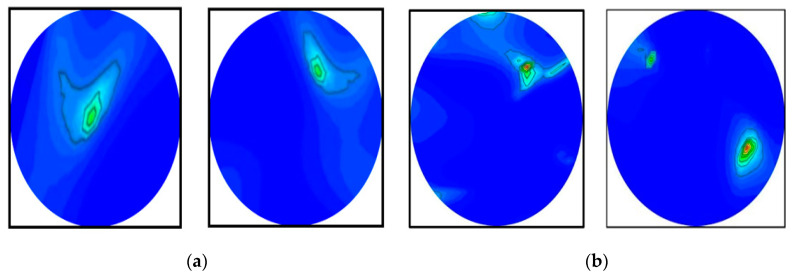

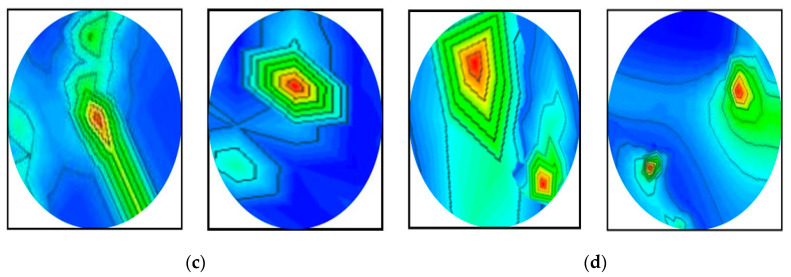
Some misclassified images by the BINet model for the raw RMW images: (**a**) non-tumor images were misclassified as a single tumor class, (**b**) single tumor images were misclassified as a non-tumor class, (**c**) single tumor images were misclassified as a double tumor class, (**d**) double tumor images were misclassified as a single tumor class.

It is evident from the classification performances in Table 8 and Table 9 that the best classification model was BINet for classifying the raw and segmented RMW brain images. The overall classification accuracy was 89.33% for the raw images and 98.33% for the segmented images, respectively. For the classification results, the confusion matrix of the BINet model for the raw RMW brain images is illustrated in Figure 15a. It is shown that there was a total of thirty-four images that were misclassified during the testing of the model. For instance, eight misclassified images are illustrated in Figure 16. It can be observed from Figure 15a that three non-tumor and fourteen double tumor images were misclassified as a single tumor class. Three double tumors and six single tumor images were misclassified as non-tumor classes, while eight single tumor images were misclassified as double tumor classes. In contrast, after segmenting the tumors, the confusion matrix of the BINet classification model is shown in Figure 15b. It can be observed from Figure 15b that only five tumor images were misclassified, and none of the non-tumor images were misclassified. One double tumor image was misclassified as a single tumor class. Additionally, one single tumor was misclassified as a non-tumor, and three single tumor images were misclassified as a double tumor class. Through the training of Self-ONNs, the optimum non-linear parameters can be learned to exploit the learning performance and attain a superior classification performance in terms of non-tumor and tumor images. However, the proposed model performed better and presented satisfactory outcomes for the segmented tumor images rather than the raw RMW tumor images. Finally, it is concluded that the segmentation technique abetted to the classification model for improving the classification performance, which is also applicable to the portable microwave brain imaging system.

## 5. Discussion about Classification Classes

In this research, we collected raw RMW brain images from the SMBI system for classifying the images by the BINet model into three classes: non-tumor, single tumor, and double tumor images. We selected the three classes due to two reasons: (i) this is our first phase of research, where we applied the M-DMAS algorithm that can reconstruct only non-tumor images and two tumor-based images, which was the limitation of the algorithm, and (ii) the fabrication recipe is another key factor for the specific tumors such as benign, malignant, meningiomas, and different categories of tumor grade, which implies the dielectric properties of the real brain tumors. In that case, proper ingredient selection was another challenge. We were unable to collect three tumor image samples and test the algorithm’s performance if the brain phantom had three tumors or different grades of tumors due to resource constraints. However, it was possible to fabricate triple tumors and different types of tumors, such as benign, malignant, meningiomas, and different grade tumors. Thus, we are designing another study as a future work for more than a double tumor or any other type of tumor (i.e., benign, malignant, meningioma, etc.).

### Future Improvement and Future Directions to Microwave Biomedical Community

We used the M-DMAS image reconstruction algorithm in this study, which can only reconstruct non-tumor images and two tumor-based images, which is one of the algorithm’s shortfalls. This is because if more than two tumors or any other types of tumors such as meningiomas, pituitary adenomas, craniopharyngiomas, etc., are formed in the brain, the algorithm will not reconstruct the images. On the other hand, in the proposed classification model, the learning outcomes of the BINet depend on the nodal operators and Q-order parameter values, which must be fixed in advance, which is another shortcoming of the model. In other words, if the right operator setting for proper learning is lacking, the learning outcomes will decrease. Moreover, there is an inadequate discrepancy due to the usage of one nodal operator set for every one of the neurons in a hidden layer. Keeping in mind the mentioned limitations, we can focus on improving the following for our future work, which will help researchers in the microwave biomedical community: (i) the implementation of a new image reconstruction algorithm that will reconstruct more than two tumors and different types of tumors, such as benign, malignant, meningiomas, etc., with high-resolution images, (ii) the implementation of a full-phase portable imaging system that can be used in a clinic or hospital, allowing for the easy use of live patients, (iii) a proper ingredient selection and quantity for fabricating the different types of tumors, (iv) an assessment of the classification performance of the proposed model for classifying different types of tumor grades by optimizing the learning parameters and Q-order, (v) computational complexity is the crucial issue for the Self-ONN model, so finding a computational complexity and inference time reduction mechanism is another research opportunity, and (vi) assessing the model by using a large multi-modal or 3D microwave brain image dataset as well as a clinical assessment with a live patient.

## 6. Conclusions

This paper presents brain tumor segmentation and classification from the portable sensors-based microwave brain imaging system through lightweight deep learning models. A lightweight MicrowaveSegNet (MSegNet) segmentation model was used to segment the brain tumors in the RMW brain images. The model can segment the target tumor regions precisely with high-resolution images and shows high segmentation performances in terms of the IoU and Dice score compared to other state-of-the-art segmentation networks. In the beginning, a compact 3D wideband nine-antenna array sensor was utilized to implement the brain imaging system framework, and then three hundred raw RMW brain tumor image samples were collected for this study. The proposed MSegNet and other nine segmentation networks were investigated and compared for verifying the segmentation performances. Among all segmentation networks, the MSegNet achieved an IoU and Dice score (DSC) of 86.92% and 93.10%, respectively, for tumor segmentation. Then, a segmented RMW brain tumor image dataset was created by applying the superimpose technique for classification purposes. After that, a lightweight BrainImageNet (BINet) classifier model was used to classify the raw and segmented RMW brain images into three classes (non-tumor, single tumor, and double tumors). The BINet uses non-linear operations to boost the network diversity and computational effectiveness and attain a superior classification performance. Furthermore, the BINet, two conventional CNNs, and three Self-ONN classification models were examined by using the raw and segmented RMW brain images, and then the classification outcomes were compared. The proposed BINet classification model showed a better perfomance compared to other models. The achieved mean accuracy, precision, recall, specificity, and F1 score of the BINet model are 89.33%, 88.74%, 88.67%, 88.61%, and 94.33%, respectively, for three classes classification using the raw RMW images, whereas they are 98.33%, 98.35%, 98.33%, 98.33%, and 99.17%, respectively, for the segmented RMW images. The high mean and low STD values ensure the efficacy of the model. The BINet model showed better classification results for the segmented tumor images rather than the original raw RMW tumor images. So, it is concluded that a combination of the MSegNet and BINet models can be used for consistently identifying the tumor(s) from the RMW brain images and this can be utilized in the portable MBI system.

## Figures and Tables

**Table 1:** The designed parameters of 3D antenna sensor.

Parameters	Value (mm)	Parameters	Value (mm)	Parameters	Value (mm)
L	53.00	b	9.34	k	4.00
W	22.00	c	4.00	t	1.00
L_1_	16.00	d	9.12	f_l_	9.50
L_2_	8.50	e	12.26	f_w_	3.00
L_3_	8.00	f	12.26	f_c_	4.24
L_4_	22.00	g	3.86	g_1_	0.50
L_5_	22.00	h	4.00	m	0.50
L_6_	3.93	i	9.22	n	1.00
a	12.26	j	9.49	..	..

**Table 2:** Comparison of the implemented system in bold with other imaging system and algorithms.

Ref.	Types of Phantom	Fabricated Tissues	Imaging System	Image Reconstruction Algorithm	No. of Detection	Application
[23]	Semi-solid heterogeneous	DURA, CSF, WM, GM	Nine-antenna-based experimental system	IC-CF-DMAS	Only one object	Microwave stroke imaging
[49]	Liquid, homogeneous	Only brain tissue	Eight-antenna-based experimental system	DBIM-TwIST	Single tumor with noisy image	Microwave tomography imaging
[22]	Semi-solid heterogeneous	Brain CSF, DURA	Single-antenna-based simulated system	Radar-based confocal	Single tumor with noisy image	Microwave brain imaging
[50]	Solid, acrylonitrile butadiene styrene (ABS)	CSF, WM, and GM	Single-antenna-based simulated system	Not stated	Single tumor with noisy image	Microwave brain imaging
[51]	Liquid, heterogeneous	Brain, CSF, fat, and muscle	Simulated imaging System	Segmentation slice-based	Single tumor with noisy image	Magnetic resonance imaging and electromagnetic imaging
[52]	Solid, acrylonitrile butadiene styrene (ABS)	Skull, CSF, brain	Two-antenna-based experimental system	EIT-based	Single tumor with blurry images	Microwave tomography imaging
[53]	Semi-solid heterogeneous	Scalp, skull, CSF	Single-antenna-based simulated system	Multi-layer time stable confocal	Single object with noisy image	Microwave brain imaging
[54]	Liquid, heterogeneous	CSF, WM, GM	Single-antenna-based experimental system	Not stated	Only one object	Microwave brain imaging
**Used Phantom**	**Semi-solid heterogeneous**	**DURA, CSF, GM, WM, fat, skin**	**Nine-antenna-based experimental imaging system**	**M-DMAS**	**Two tumors with clear image**	**Sensor-based Microwave brain tumor imaging system (SMBIS)**

**Table 3:** Dataset description for training, testing, and validation.

Dataset	Number of Original Images	Image Classes	Training Dataset			
			Number of Images per Class	Augmented Train Images per Fold	Testing Images per Fold	Validation Image per Fold
Raw RMW brain image samples	300	Non-tumor	100	1980	20	16
		Single tumor	100	2008	20	16
		Double tumors	100	2012	20	16
**Total**			**300**	**6000**	**60**	**48**

**Table 4:** Hyper-parameters for all segmentation models.

Parameter’s Name	Assigned Value	Parameter’s Name	Assigned Value
Input channels	3	Output channels	1
Batch size	8	Optimizer	Adam
Learning rate (LR)	0.0005	Loss type	Dice loss
Maximum number of epochs	30	Epochs patience	10
Maximum epochs stop	15	Learning factor	0.2
Initial feature	32	Number of folds	5

**Table 5:** Hyper-parameters for all classification models.

Parameter’s Name	Assigned Value	Parameter’s Name	Assigned Value
Input channels	3	Q order	1 for CNN, 3 for Self-ONNs
Batch size	16	Optimizer	Adam
Learning rate (LR)	0.0005	Stop criteria	Loss
Maximum number of epochs	30	Epochs patience	5
Maximum epochs stop	10	Learning factor	0.2
Image size	224	Number of folds	5

**Table 6:** Performance evaluation matrices of all segmentation models. Bold represents the best performing model.

Network Model Name	Accuracy (%)	IoU (%)	Dice Score (%)	Loss
U-net	99.96	85.72	91.58	0.1127
Modified Unet (M-Unet)	99.96	86.47	92.20	0.1086
Keras Unet (K-Unet)	99.96	86.01	91.91	0.1156
MultiResUnet	99.96	86.55	92.20	0.1064
ResNet50	99.95	86.43	92.13	0.1121
DenseNet161	99.95	85.62	91.59	0.1145
ResNet152 FPN	99.94	82.86	89.58	0.1312
DenseNet121 FPN	99.95	83.30	89.91	0.1318
nnU-net	99.96	84.95	92.85	0.1112
**Proposed MSegNet**	**99.97**	**86.92**	**93.10**	**0.1010**

**Table 7:** Computational complexity comparison of the proposed model with Unet based models.

Network Model Name	Parameters (M)	Training Time (Second/Fold)	Inference Time (Second/Image)
U-net	30	480	0.025
Modified Unet (M-Unet)	28	440	0.023
Keras Unet (K-Unet)	30	490	0.026
MultiResUnet	25	425	0.02
ResNet50	25	420	0.023
DenseNet161	28.5	450	0.033
ResNet152 FPN	40	720	0.05
DenseNet121 FPN	20	410	0.021
nnU-net	18	340	0.015
**Proposed MSegNet**	**8**	**305**	**0.007**

**Table 8:** Statistical classification results of all models for the raw RMW brain images. Bold represents the best performing model.

Image Type	Network Model Name	Overall		Weighted								*p*-Value
		Accuracy (A)		Precession (P)		Recall (R)		Specificity (S)		F1 Score (F_s_)		
		Mean	STD	Mean	STD	Mean	STD	Mean	STD	Mean	STD	
Raw RMW Brain Images	Vanilla CNN6L	84.33	4.11	84.17	4.13	84.33	4.11	92.17	3.04	84.06	4.14	<0.05
	Vanilla CNN8L	85.33	4.00	85.62	3.97	85.33	4.00	92.67	2.95	85.14	4.03	<0.05
	Self-ONN4L	85.00	4.04	84.91	4.05	85.00	4.04	92.50	2.98	84.87	4.06	<0.05
	Self-ONN4L1DN	87.00	3.81	87.05	3.80	87.00	3.81	93.50	2.79	86.95	3.81	<0.05
	Self-ONN6L	87.00	3.81	86.85	3.82	87.00	3.81	93.50	2.79	86.82	3.83	<0.05
	**Proposed** **BINet**	**89.33**	**3.49**	**88.74**	**3.58**	**88.67**	**3.59**	**94.33**	**2.62**	**88.61**	**3.59**	**<0.05**

**Table 9:** Statistical classification results of all models for the segmented RMW brain images. Bold represents the best performing model.

Image Type	Network Model Name	Overall		Weighted								*p*-Value
		Accuracy (A)		Precession (P)		Recall (R)		Specificity (S)		F1 Score (F_s_)		
		Mean	STD	Mean	STD	Mean	STD	Mean	STD	Mean	STD	
Segmented RMW Brain Images	Vanilla CNN6L	95.00	2.47	94.98	2.47	95.00	2.47	97.50	1.77	94.96	2.48	<0.05
	Vanilla CNN8L	95.67	2.30	95.77	2.28	95.67	2.30	97.83	1.65	95.65	2.31	<0.05
	Self-ONN4L	94.00	2.69	93.96	2.70	94.00	2.69	97.00	1.93	93.96	2.70	<0.05
	Self-ONN4L1DN	96.33	2.13	96.41	2.11	97.00	1.93	98.17	1.52	97.00	1.93	<0.05
	Self-ONN6L	96.67	2.03	96.79	1.99	96.67	2.03	98.33	1.45	96.66	2.03	<0.05
	**Proposed** **BINet**	**98.33**	**1.45**	**98.35**	**1.44**	**98.33**	**1.45**	**99.17**	**1.03**	**98.33**	**1.45**	**<0.05**

## Data Availability

Data are not publicly available due to privacy restrictions.

## References

[1] Tracy Wyant R.A. (2021). Cynthia Ogoro. Key Statistics for Brain and Spinal Cord Tumors.

[2] Ahmad H.A., Yu H.J., Miller C.G. (2014). Medical imaging modalities. Medical Imaging in Clinical Trials.

[3] Frangi A.F., Tsaftaris S.A., Prince J.L. (2018). Simulation and synthesis in medical imaging. IEEE Trans. Med. Imaging.

[4] Tariq M., Siddiqi A.A., Narejo G.B., Andleeb S. (2019). A cross sectional study of tumors using bio-medical imaging modalities. Curr. Med. Imaging.

[5] Adamson E.B., Ludwig K.D., Mummy D.G., Fain S.B. (2017). Magnetic resonance imaging with hyperpolarized agents: Methods and applications. Phys. Med. Biol..

[6] Cazzato R.L., Garnon J., Shaygi B., Koch G., Tsoumakidou G., Caudrelier J., Addeo P., Bachellier P., Namer I.J., Gangi A. (2018). PET/CT-guided interventions: Indications, advantages, disadvantages and the state of the art. Minim. Invasive Ther. Allied Technol..

[7] Chakraborty S., Chatterjee S., Ashour A.S., Mali K., Dey N. (2018). Intelligent computing in medical imaging: A study. Advancements in Applied Metaheuristic Computing.

[8] Dougeni E., Faulkner K., Panayiotakis G. (2012). A review of patient dose and optimisation methods in adult and paediatric CT scanning. Eur. J. Radiol..

[9] Jacobs M.A., Ibrahim T.S., Ouwerkerk R. (2007). MR imaging: Brief overview and emerging applications. Radiographics.

[10] Jones K.M., Michel K.A., Bankson J.A., Fuller C.D., Klopp A.H., Venkatesan A.M. (2018). Emerging magnetic resonance imaging technologies for radiation therapy planning and response assessment. Int. J. Radiat. Oncol. Biol. Phys..

[11] Alqadami A.S., Bialkowski K.S., Mobashsher A.T., Abbosh A.M. (2018). Wearable electromagnetic head imaging system using flexible wideband antenna array based on polymer technology for brain stroke diagnosis. IEEE Trans. Biomed. Circuits Syst..

[12] Stancombe A.E., Bialkowski K.S., Abbosh A.M. (2019). Portable microwave head imaging system using software-defined radio and switching network. IEEE J. Electromagn. RF Microw. Med. Biol..

[13] Tobon Vasquez J.A., Scapaticci R., Turvani G., Bellizzi G., Joachimowicz N., Duchêne B., Tedeschi E., Casu M.R., Crocco L., Vipiana F. (2019). Design and experimental assessment of a 2D microwave imaging system for brain stroke monitoring. Int. J. Antennas Propag..

[14] Hossain A., Islam M.T., Almutairi A.F., Singh M.S.J., Mat K., Samsuzzaman M. (2020). An octagonal ring-shaped parasitic resonator based compact ultrawideband antenna for microwave imaging applications. Sensors.

[15] Hossain A., Islam M.T., Chowdhury M.E., Samsuzzaman M. (2020). A grounded coplanar waveguide-based slotted inverted delta-shaped wideband antenna for microwave head imaging. IEEE Access.

[16] Hossain A., Islam M.T., Islam M., Chowdhury M.E., Rmili H., Samsuzzaman M. (2020). A Planar Ultrawideband Patch Antenna Array for Microwave Breast Tumor Detection. Materials.

[17] Mobashsher A., Bialkowski K., Abbosh A., Crozier S. (2016). Design and experimental evaluation of a non-invasive microwave head imaging system for intracranial haemorrhage detection. PLoS ONE.

[18] Mobashsher A.T., Abbosh A.M., Wang Y. (2014). Microwave system to detect traumatic brain injuries using compact unidirectional antenna and wideband transceiver with verification on realistic head phantom. IEEE Trans. Microw. Theory Tech..

[19] Mohammed B.J., Abbosh A.M., Mustafa S., Ireland D. (2013). Microwave system for head imaging. IEEE Trans. Instrum. Meas..

[20] Salleh A., Yang C., Alam T., Singh M., Samsuzzaman M., Islam M. (2020). Development of microwave brain stroke imaging system using multiple antipodal vivaldi antennas based on raspberry Pi technology. J. Kejuruterran.

[21] Fedeli A., Estatico C., Pastorino M., Randazzo A. (2020). Microwave detection of brain injuries by means of a hybrid imaging method. IEEE Open J. Antennas Propag..

[22] Inum R., Rana M., Shushama K.N., Quader M. (2018). EBG based microstrip patch antenna for brain tumor detection via scattering parameters in microwave imaging system. Int. J. Biomed. Imaging.

[23] Islam M.S., Islam M.T., Hoque A., Islam M.T., Amin N., Chowdhury M.E. (2021). A portable electromagnetic head imaging system using metamaterial loaded compact directional 3D antenna. IEEE Access.

[24] Rezaeieh S.A., Zamani A., Abbosh A. (2014). 3-D wideband antenna for head-imaging system with performance verification in brain tumor detection. IEEE Antennas Wirel. Propag. Lett..

[25] Rokunuzzaman M., Ahmed A., Baum T.C., Rowe W.S. (2019). Compact 3-D antenna for medical diagnosis of the human head. IEEE Trans. Antennas Propag..

[26] Gerazov B., Conceicao R.C. (2017). Deep learning for tumour classification in homogeneous breast tissue in medical microwave imaging. Proceedings of IEEE EUROCON 2017-17th International Conference on Smart Technologies.

[27] Khoshdel V., Asefi M., Ashraf A., LoVetri J. (2020). Full 3D microwave breast imaging using a deep-learning technique. J. Imaging.

[28] Rana S.P., Dey M., Tiberi G., Sani L., Vispa A., Raspa G., Duranti M., Ghavami M., Dudley S. (2019). Machine learning approaches for automated lesion detection in microwave breast imaging clinical data. Sci. Rep..

[29] Salucci M., Polo A., Vrba J. (2021). Multi-step learning-by-examples strategy for real-time brain stroke microwave scattering data inversion. Electronics.

[30] Shah P., Moghaddam M. (2017). Super resolution for microwave imaging: A deep learning approach. Proceedings of 2017 IEEE International Symposium on Antennas and Propagation & USNC/URSI National Radio Science Meeting.

[31] Shao W., Du Y. (2020). Microwave imaging by deep learning network: Feasibility and training method. IEEE Trans. Antennas Propag..

[32] Bakas S., Reyes M., Jakab A., Bauer S., Rempfler M., Crimi A., Shinohara R., Berger C., Ha S., Rozycki M. (2018). Identifying the best machine learning algorithms for brain tumor segmentation, progression assessment, and overall survival prediction in the BRATS challenge. arXiv.

[33] Isensee F., Jaeger P.F., Kohl S.A., Petersen J., Maier-Hein K.H. (2021). nnU-Net: A self-configuring method for deep learning-based biomedical image segmentation. Nat. Methods.

[34] Ronneberger O., Fischer P., Brox T. (2015). U-net: Convolutional networks for biomedical image segmentation. Proceedings of International Conference on Medical Image Computing and Computer-Assisted Intervention.

[35] Cheng G., Ji H. (2020). Adversarial Perturbation on MRI Modalities in Brain Tumor Segmentation. IEEE Access.

[36] Ding Y., Chen F., Zhao Y., Wu Z., Zhang C., Wu D. (2019). A stacked multi-connection simple reducing net for brain tumor segmentation. IEEE Access.

[37] Aboelenein N.M., Songhao P., Koubaa A., Noor A., Afifi A. (2020). HTTU-Net: Hybrid two track U-net for automatic brain tumor segmentation. IEEE Access.

[38] Hu K., Gan Q., Zhang Y., Deng S., Xiao F., Huang W., Cao C., Gao X. (2019). Brain tumor segmentation using multi-cascaded convolutional neural networks and conditional random field. IEEE Access.

[39] Chen W., Liu B., Peng S., Sun J., Qiao X. (2019). S3D-UNet: Separable 3D U-Net for brain tumor segmentation. Proceedings of International MICCAI Brainlesion Workshop.

[40] Noreen N., Palaniappan S., Qayyum A., Ahmad I., Imran M., Shoaib M. (2020). A deep learning model based on concatenation approach for the diagnosis of brain tumor. IEEE Access.

[41] Ding Y., Li C., Yang Q., Qin Z., Qin Z. (2019). How to improve the deep residual network to segment multi-modal brain tumor images. IEEE Access.

[42] Hao J., Li X., Hou Y. (2020). Magnetic resonance image segmentation based on multi-scale convolutional neural network. IEEE Access.

[43] Kumar R.L., Kakarla J., Isunuri B.V., Singh M. (2021). Multi-class brain tumor classification using residual network and global average pooling. Multimed. Tools Appl..

[44] Devecioglu O.C., Malik J., Ince T., Kiranyaz S., Atalay E., Gabbouj M. (2021). Real-time glaucoma detection from digital fundus images using Self-ONNs. IEEE Access.

[45] Kiranyaz S., Malik J., Abdallah H.B., Ince T., Iosifidis A., Gabbouj M. (2021). Self-organized operational neural networks with generative neurons. Neural Netw..

[46] Hossain A., Islam M.T., Islam M.S., Chowdhury M.E., Almutairi A.F., Razouqi Q.A., Misran N. (2021). A YOLOv3 Deep Neural Network Model to Detect Brain Tumor in Portable Electromagnetic Imaging System. IEEE Access.

[47] Mobashsher A., Abbosh A. (2014). Three-dimensional human head phantom with realistic electrical properties and anatomy. IEEE Antennas Wirel. Propag. Lett..

[48] Hossain A., Islam M.T., Almutairi A.F. (2022). A deep learning model to classify and detect brain abnormalities in portable microwave based imaging system. Sci. Rep..

[49] Karadima O., Rahman M., Sotiriou I., Ghavami N., Lu P., Ahsan S., Kosmas P. (2020). Experimental validation of microwave tomography with the DBIM-TwIST algorithm for brain stroke detection and classification. Sensors.

[50] Joachimowicz N., Duchêne B., Conessa C., Meyer O. (2018). Anthropomorphic breast and head phantoms for microwave imaging. Diagnostics.

[51] Wood S., Krishnamurthy N., Santini T., Raval S.B., Farhat N., Holmes J.A., Ibrahim T.S. (2018). Correction: Design and fabrication of a realistic anthropomorphic heterogeneous head phantom for MR purposes. PLoS ONE.

[52] Zhang J., Yang B., Li H., Fu F., Shi X., Dong X., Dai M. (2017). A novel 3D-printed head phantom with anatomically realistic geometry and continuously varying skull resistivity distribution for electrical impedance tomography. Sci. Rep..

[53] Pokorny T., Vrba D., Tesarik J., Rodrigues D.B., Vrba J. (2019). Anatomically and dielectrically realistic 2.5 D 5-layer reconfigurable head phantom for testing microwave stroke detection and classification. Int. J. Antennas Propag..

[54] Li C.-W., Hsu A.-L., Huang C.-W.C., Yang S.-H., Lin C.-Y., Shieh C.-C., Chan W.P. (2020). Reliability of synthetic brain MRI for assessment of ischemic stroke with phantom validation of a relaxation time determination method. J. Clin. Med..

[55] Chang S.W., Liao S.W. (2019). KUnet: Microscopy image segmentation with deep unet based convolutional networks. Proceedings of 2019 IEEE International Conference on Systems, Man and Cybernetics (SMC).

[56] Ronneberger O., Fischer P., Brox T. (2019). U-Net: Convolutional networks for biomedical image segmentation. arXiv.

[57] Kiranyaz S., Ince T., Iosifidis A., Gabbouj M. (2020). Operational neural networks. Neural Comput. Appl..

[58] Kiranyaz S., Malik J., Abdallah H.B., Ince T., Iosifidis A., Gabbouj M. (2021). Exploiting heterogeneity in operational neural networks by synaptic plasticity. Neural Comput. Appl..

[59] Malik J., Kiranyaz S., Gabbouj M. (2020). Operational vs. convolutional neural networks for image denoising. arXiv.

[60] Malik J., Kiranyaz S., Gabbouj M. (2021). Self-organized operational neural networks for severe image restoration problems. Neural Netw..

[61] Rahman T., Khandakar A., Qiblawey Y., Tahir A., Kiranyaz S., Kashem S.B.A., Islam M.T., Al Maadeed S., Zughaier S.M., Khan M.S. (2021). Exploring the effect of image enhancement techniques on COVID-19 detection using chest X-ray images. Comput. Biol. Med..

